# A Practical Strategy for Exploring the Pharmacological Mechanism of Luteolin Against COVID-19/Asthma Comorbidity: Findings of System Pharmacology and Bioinformatics Analysis

**DOI:** 10.3389/fimmu.2021.769011

**Published:** 2022-01-07

**Authors:** Yi-Zi Xie, Chen-Wen Peng, Zu-Qing Su, Hui-Ting Huang, Xiao-Hong Liu, Shao-Feng Zhan, Xiu-Fang Huang

**Affiliations:** ^1^ The First Affiliated Hospital of Guangzhou University of Chinese Medicine, Guangzhou, China; ^2^ The First Clinical Medical College of Guangzhou University of Chinese Medicine, Guangzhou, China; ^3^ Guangdong Provincial Hospital of Chinese Medicine, The Second Clinical College of Guangzhou University of Chinese Medicine, Guangzhou, China; ^4^ Lingnan Medical Research Center of Guangzhou University of Chinese Medicine, Guangzhou, China

**Keywords:** luteolin, COVID-19, asthma, comorbidity, system pharmacology, bioinformatics analysis

## Abstract

Asthma patients may increase their susceptibility to SARS-CoV-2 infection and the poor prognosis of coronavirus disease 2019 (COVID-19). However, anti-COVID-19/asthma comorbidity approaches are restricted on condition. Existing evidence indicates that luteolin has antiviral, anti-inflammatory, and immune regulation capabilities. We aimed to evaluate the possibility of luteolin evolving into an ideal drug and explore the underlying molecular mechanisms of luteolin against COVID-19/asthma comorbidity. We used system pharmacology and bioinformatics analysis to assess the physicochemical properties and biological activities of luteolin and further analyze the binding activities, targets, biological functions, and mechanisms of luteolin against COVID-19/asthma comorbidity. We found that luteolin may exert ideal physicochemical properties and bioactivity, and molecular docking analysis confirmed that luteolin performed effective binding activities in COVID-19/asthma comorbidity. Furthermore, a protein–protein interaction network of 538 common targets between drug and disease was constructed and 264 hub targets were obtained. Then, the top 6 hub targets of luteolin against COVID-19/asthma comorbidity were identified, namely, TP53, AKT1, ALB, IL-6, TNF, and VEGFA. Furthermore, the enrichment analysis suggested that luteolin may exert effects on virus defense, regulation of inflammation, cell growth and cell replication, and immune responses, reducing oxidative stress and regulating blood circulation through the Toll-like receptor; MAPK, TNF, AGE/RAGE, EGFR, ErbB, HIF-1, and PI3K–AKT signaling pathways; PD-L1 expression; and PD-1 checkpoint pathway in cancer. The possible “dangerous liaison” between COVID-19 and asthma is still a potential threat to world health. This research is the first to explore whether luteolin could evolve into a drug candidate for COVID-19/asthma comorbidity. This study indicated that luteolin with superior drug likeness and bioactivity has great potential to be used for treating COVID-19/asthma comorbidity, but the predicted results still need to be rigorously verified by experiments.

## Introduction

The outbreak of coronavirus disease 2019 (COVID-19) emerged in December 2019 and quickly spread worldwide. It is recognized that severe acute respiratory syndrome coronavirus 2 (SARS-CoV-2) binds to angiotensin-converting enzyme 2 (ACE2) to initiate infectious procedures (1, 2). As SARS-CoV-2 has the characteristics of a high fatality rate, COVID-19 affecting global health and economy has become a serious public health emergency (1, 3). Although efforts to develop vaccines and therapeutic drugs to combat COVID-19 are proceeding at the fastest scale, COVID-19 still has a negative impact on human health (4–6). The access to aerosol is very restrictive in resource-poor countries for the COVID-19 pandemic, resulting in exacerbating the incidence and development of asthma (4). There are good reasons to worry about the possible “dangerous liaison” between COVID-19 and asthma. SARS-CoV-2 infection can cause a series of respiratory problems and even progress to respiratory failure with acute respiratory distress syndrome (7). Asthma patients with the persistence of impaired innate immune responses are more susceptible to releasing lower levels of INF during viral respiratory infections (8–10). Moreover, a cohort study finds that children with COVID-19/asthma comorbidity show more serious disease progression and a single-center retrospective propensity-matched analysis proves that patients infected with COVID-19 have a higher prevalence of asthma (11, 12). In addition, a study recruiting 493,000 patients from the UK Biobank confirms that adults with asthma have a higher risk of suffering from COVID-19 and a nationwide cohort study performed by Koreans proves that asthma contributes to increased susceptibility to SARS-CoV-2 infection and poor prognosis of COVID-19 (13, 14). Therefore, we need to raise the warning of the possibility that asthma patients have a great potential to be distinctly vulnerable to developing COVID-19 comorbidity and experiencing serious clinical consequences.

Inhaled corticosteroids (ICS) as the first-line treatment for asthma are thought to be an immunosuppressive agent, which might enhance the possibility of upper respiratory infection in asthma (15, 16). Moreover, research reveals that ICS are harmful to antiviral innate immune response (17). Jordan et al. conduct a study incorporating 818,490 asthma patients that estimates the relationship between the fatality risk of COVID-19 and ICS treatment and find that ICS intervention may cause damage and suboptimal outcome to asthma/mild COVID-19 comorbidity patients (18). A recovery trial corrected that systemic glucocorticoid treatment for patients with mild COVID-19 increases the risk of death, and another study finds that treatment with glucocorticoid shows no clinical benefit for COVID-19-caused mild to moderate acute respiratory distress syndrome (11, 19, 20). The above results indicate that glucocorticoid for COVID-19/asthma comorbidity patients requires cautious interpretation, and thus, safer and more effective interventions or adjunctive interventions are likely to be afforded for such patients.

**Figure 1:**
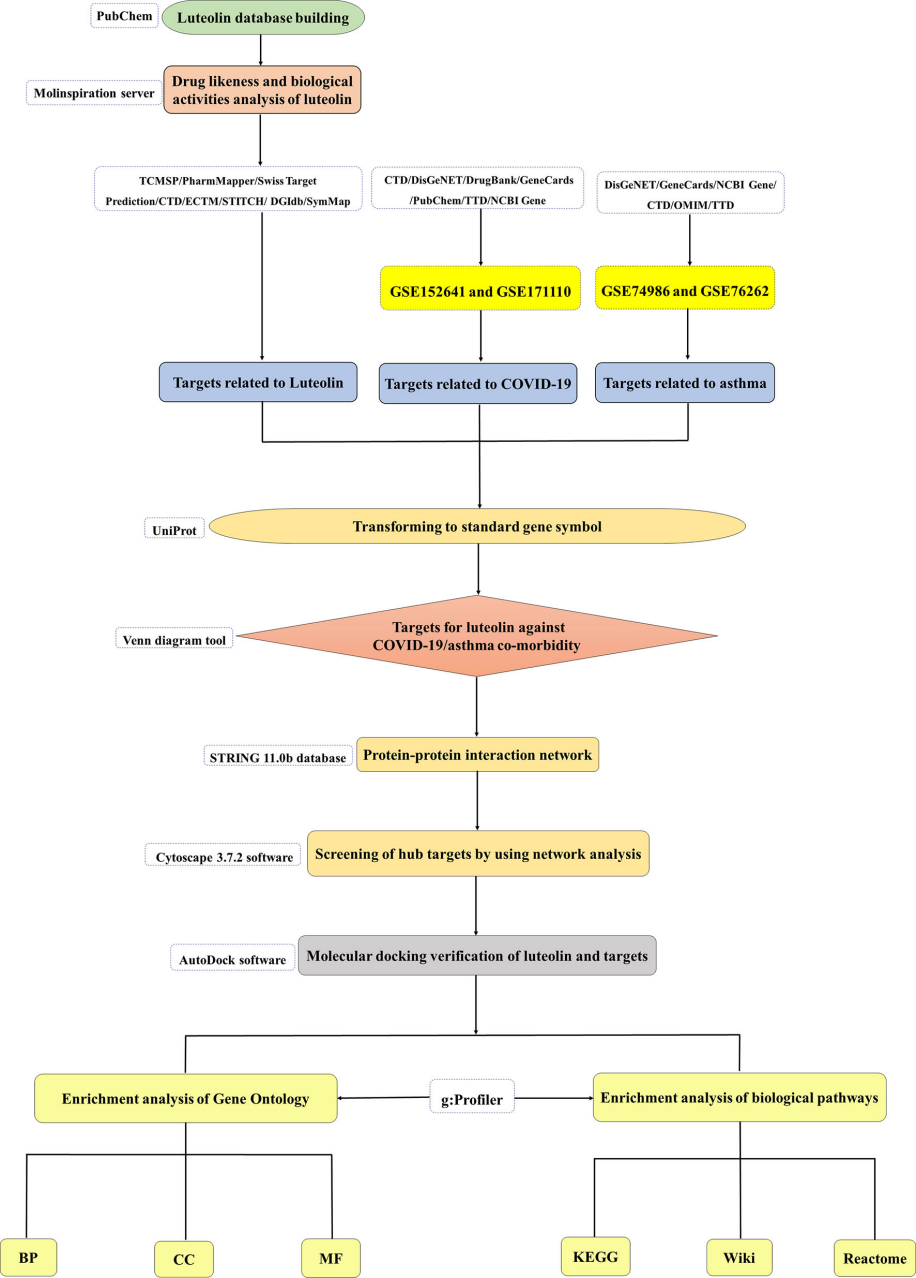
The flow diagram of this research showing a pragmatic strategy for identifying the pharmacological mechanisms of luteolin against coronavirus disease 2019 (COVID-19)/asthma comorbidity based on system pharmacology and bioinformatics analysis.

Luteolin has shown broad antiviral and anti-inflammatory capabilities (21–24). Surprisingly, studies have confirmed that luteolin can specifically bind to the surface spike protein of SARS-CoV-2 to block viral entrance into the host cells and inhibit the expression of SARS-CoV 3CL protease (25, 26). Furthermore, it is exciting to find that luteolin and the luteolin structural analog eriodictyol have great potential to be the inhibitors of COVID-19 (19, 27). Luteolin can inhibit the cytokine storm caused by the production of IL-1β and histamine by mast cells stimulated by SARS-CoV-2 (28–31). Its novel structural analogs methoxyluteolin and 3′,4′,5,7-tetramethoxyluteolin also inhibit human mast cells to perform anti-inflammatory effect (32, 33). Meanwhile, appropriate luteolin formulations may also prevent or reduce brain fog associated with long COVID-19 syndrome (34). Luteolin is also considered to attenuate bronchoconstriction and airway hyperreactivity, thus has the potential to become a promising therapeutic intervention against asthma (35). To our knowledge, the therapeutic targets and molecular mechanisms of luteolin against COVID-19 in asthma patients have not been previously explored. Based on this, we used system pharmacology and bioinformatics analysis to assess the drug likeness and bioactivity of luteolin and analyze the targets and signaling pathways of luteolin against COVID-19/asthma comorbidity. The flow diagram of our research is shown in **Figure 1**.

## Materials and Methods

### Ethics Statement

The data were obtained from open-source databases, and thus, ethics committee approval was not applicable in this study.

### Luteolin Database Building

PubChem (https://pubchem.ncbi.nlm.nih.gov/) comprises a wide range of chemical information from 750 data sources (36). The 2D structure, 3D structure, InChI, and canonical SMILES profiles of luteolin were obtained from PubChem (36).

### Analyses of Physicochemical Properties and Biological Activities

The Molinspiration server (https://www.molinspiration.com/), an open-source and highly efficient chemoinformatics tool, can carry out molecular manipulation and processing. Thus, the Molinspiration server was used to evaluate the molecular descriptors, drug likeness, and bioactivity of luteolin (37). The standard SMILES profile of luteolin was uploaded to “Calculation of Molecular Properties and Bioactivity Score” section. The drug likeness analyzed based on the Lipinski’s rule of five comprised the following parameters: logP, molecular weight (MW), number of hydrogen bond acceptors (*n*-ON), and number of hydrogen bond donors (*n*-OHNH) (38). The biological activity analysis of luteolin included G-protein-coupled receptor (GPCR) ligand, ion channel modulator, kinase inhibitor, nuclear receptor ligand, protease inhibitor, and enzyme inhibitor (37–40). The additional percentage of absorption (%ABS) value was calculated according to the following formula: %ABS = 109 − [0.345 × TPSA] (38).

### Fishing of Luteolin-Related Targets

Different types of pharmacological targets related to luteolin were collected from the following databases: 1) Traditional Chinese Medicine Systems Pharmacology Database and Analysis Platform (TCMSP, http://tcmspw.com/) (41), 2) PharmMapper (http://www.lilabecust.cn/pharmmapper/) (42), 3) Swiss Target Prediction (http://www.swisstargetprediction.ch/) (43), 4) Chemical Association Networks (STITCH, http://stitch.embl.de/) (44), 5) Comparative Toxicogenomics Database (CTD, http://ctdbase.org/) (45), 6) Drug Gene Interaction Database (DGIdb, https://www.dgidb.org/) (46), 7) Encyclopedia of Traditional Chinese Medicine (ECTM, http://www.tcmip.cn/ETCM/) (47), and 8) Symptom Mapping (SymMap, https://www.Symmap.org/) (48). The target proteins were transformed to standard gene symbols by using the UniProt database (https://www.uniprot.org/) with the limitation of “*Human species*”.

### Collection of COVID-19 or Asthma-Related Targets

The COVID-19-related targets were identified from differentially expressed genes (DEGs) by analyzing available transcriptomic RNA-seq data of COVID-19 (GSE152641 and GSE171110) from the Gene Expression Omnibus database (GEO, https://www.ncbi.nlm.nih.gov/geo) (49). The “limma” in R (version 3.6.2, https://www.r-project.org/) was applied to access the profile of DEGs, which must fit the screening criteria of adjusted *P*-value <0.05 and |log2FC| >1 (50). DEGs were visualized by volcano plots, which were drawn by “ggpubr” and “ggthemes” of R-language package. Moreover, the COVID-19-related targets were also gathered from seven open-source databases listed as follows: 1) CTD (http://ctdbase.org/), 2) DisGeNET (http://www.disgenet.org), 3) DrugBank (https://go.drugbank.com/) (51), 4) GeneCards (https://www.genecards.org/) (52), 5) PubChem, 6) Therapeutic Target Database (TTD, http://db.idrblab.net/) (53), and 7) NCBI Gene (https://www.ncbi.nlm.nih.gov/).

As for asthma-related targets, we first analyzed the DEGs from the GSE74986 and GSE76262 datasets from the GEO database, which were also assessed by the “limma” package of R-language Bioconductor with the criteria of adjusted *P*-value <0.05 and |log2FC| >1 (50). Additionally, targets related to asthma were also acquired by exploring the following six databases: 1) CTD, 2) DisGeNET, 3) GeneCards, 4) Online Mendelian Inheritance in Man (OMIM, https://omim.org/) (54), 5) TTD, and 6) NCBI Gene.

### Targets of Luteolin Against COVID-19/Asthma Comorbidity Acquisition

The overlapping targets between luteolin, asthma, and COVID-19 were further obtained by using the Venn diagram tool (http://bioinformatics.psb.ugent.be/webtools/Venn/) and Microsoft Excel. The intersection between luteolin-related targets and COVID-19/asthma-related targets was the final targets of luteolin against COVID-19/asthma comorbidity.

### Analyses of the Protein–Protein Interaction Network and Hub Targets

The protein–protein interaction (PPI) network helps to better understand the biological mechanisms involved in target-related pathogenesis at the protein level. Thus, the STRING 11.0b database (https://string-db.org/) was used to construct the PPI network and receive hub targets. The organism was set to “*Homo sapiens*” and the minimum required interaction score was 0.4 (55). Subsequently, the PPI network was visualized and analyzed by Cytoscape 3.7.2 software (https://cytoscape.org/). The degree values in the PPI network were calculated by using the NetworkAnalyzer plugin of Cytoscape 3.7.2 software. Then, targets with degree values higher than the median were filtered as hub targets (56).

### Enrichment Analyses for Hub Targets

Enrichment analyses of Gene Ontology (GO) (including molecular function, cellular component, and biological process) and biological pathways (including KEGG pathways, Reactome pathways, and Wiki pathways) of hub targets were carried out through g:Profiler (https://biit.cs.ut.ee/gprofiler/gost) (57). The organism was set to “*Homo sapiens*” and a term with adjusted *P*-value <0.05 was considered significantly enriched. The GO terms or pathway terms with smaller adjusted *P*-values were believed to have more potent effects on fighting COVID-19/asthma comorbidity, and the top 30 GO terms and pathway terms were illustrated in the results ranked by adjusted *P*-value from low to high.

### Molecular Docking Verification of Luteolin and Targets

The protein structures of targets were downloaded from the PDB database (https://www.rcsb.org/) (58). The 3D structure of luteolin was provided by the PubChem database. Pymol (https://pymol.org/2/) and AutoDock software (Vina 1.5.6, http://autodock.scripps.edu/) were applied to extract the ligand from the target protein, then the original ligand to the active site of the complex was redocked, and the conformation of the original ligand was compared with the conformation of the ligand after docking (59). Root mean square deviation (RMSD) would be considered that the docking method is reliable when RMSD <2 (60). Furthermore, the water molecules were removed, hydrogen atoms were added, and the spatially active sites of ligand molecules docking in the target protein compound were determined for docking preparation.

Luteolin was docked with targets, including spike (S)-protein, receptor-binding domain (RBD), main protease (Mpro), ACE2, transmembrane protease serine 2 (TMPRSS2), cluster differentiation 147 (CD147), and the top 6 hub targets predicted by the PPI network through the AutoDock software. The position coordinates of the ligand in the target protein were defined as active pocket and the spacing was set to 0.375. Then, “Run AutoDock” was clicked to perform the molecular docking and binding energy <0 was supposed that luteolin could spontaneously bind to the targets (61).

## Results

### Analysis of the Physicochemical Properties of Luteolin

The evaluation of drug likeness is of vital importance in the production and upgrading of drug entities, and we first predicted the physicochemical properties of luteolin according to the Lipinski’s rule of five. The criteria of the Lipinski’s rule of five are as follows: logP ≤5, MW ≤500 Da, *n*-ON ≤10, and *n*-OHNH ≤5 (38). In addition, the topological polar surface area (TPSA) value is a key indicator for evaluating and predicting the oral bioavailability of molecular compounds, and the TPSA value of ≤140 Å represents good oral bioavailability (37, 62–64). %ABS value calculated according to TPSA value between 67% and 83% means an ideal oral bioavailability (37, 62–64). Surprisingly, the results showed that luteolin met the criteria of logP = 1.97 < 5, MW = 286.24 < 500 Da, *n*-ON = 6 < 10, and *n*-OHNH = 4 <5, and the value of TPSA at 111.12 Å < 140 and %ABS at 70.66 that ranged from 57.95% to 78.98% were at the range of ideal oral bioavailability as shown in **Table 1**.

**Table 1:** Physicochemical properties of luteolin evaluated by Molinspiration.

Compound	%ABS	miLogP	TPSA (Å)	*n*-atoms	MW	*n*-ON	*n*-OHNH	*n*-violations	*n*-rotb	MV
**Standard criteria**		<5			<500	<10	<5	≤1	≤10	
**Luteolin**	70.66	1.97	111.12	21	286.24	6	4	0	1	232.07

%ABS, percentage of absorption; miLogP, logarithm of partition coefficient between n-octanol and water; TPSA, topological polar surface area; n-atoms, number of atoms; MW, molecular weight; n-ON, number of hydrogen bond acceptors; n-OHNH, number of hydrogen bond donors; n violations, number of Lipinski’s rule-of-five violation; n-rotb, number of rotatable bonds; MV, molecular volume.

### Bioactivity Prediction of Luteolin

As summarized in **Table 2**, the physiological role of luteolin may be associated with a variety of mechanisms, including possible interactions with GPCR ligands, ion channel modulator, kinase inhibitor, nuclear receptor ligand, protease inhibitor, and enzyme inhibitor. Moreover, luteolin exhibited promising kinase inhibitor, nuclear receptor ligand, and enzyme inhibitor affinities with bioactivity scores greater than 0.2 > 0, while it showed moderate GPCR ligand, ion channel modulator, and protease inhibitor affinities with bioactivity values between −0.50 and 0.00. The results indicated that luteolin had better nuclear receptor ligand affinity (nuclear receptor ligand > enzyme inhibitor > kinase inhibitor > GPCR ligand > ion channel modulation > protease inhibitor).

**Table 2:** Bioactivity scores of luteolin based on Molinspiration cheminformatics.

Compound	GPCR ligand	Ion channel modulator	Kinase inhibitor	Nuclear receptor ligand	Protease inhibitor	Enzyme inhibitor
**Luteolin**	−0.02	−0.07	0.26	0.39	−0.22	0.28

Bioactivity score of >0 represented promising activity, bioactivity score between −5.00 and 0.00 represented moderate activity, and bioactivity score of ≤5.0 represented no activity.

### Target Identification of Luteolin and COVID-19/Asthma Comorbidity

**Figure 2:**
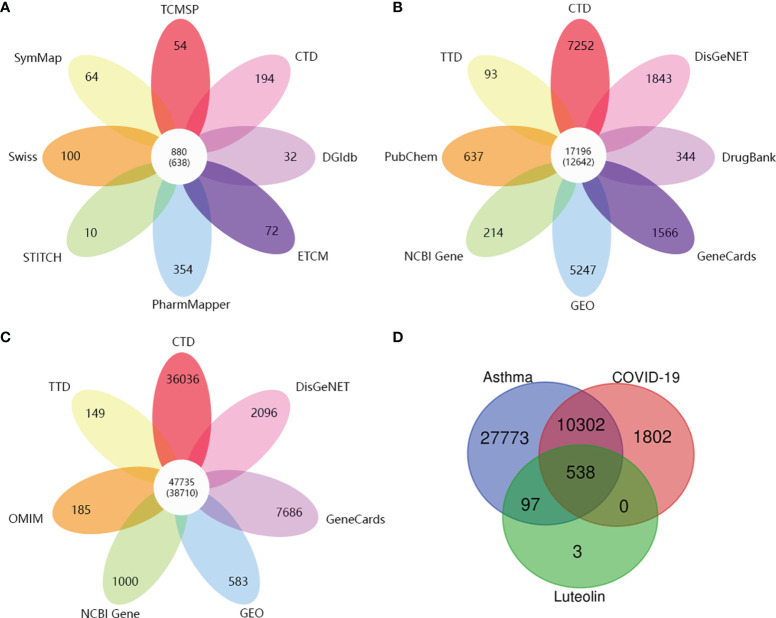
**(A)** The number of target genes related to luteolin from eight open-source databases. **(B)** The number of target genes related to COVID-19 from eight open-source databases. **(C)** The number of asthma-related target genes from seven open-source databases. **(D)** Venn diagram depicting common target genes between COVID-19, asthma, and luteolin.

Eight open-source databases were used to obtain the targets related to luteolin, namely, TCMSP (54), PharmMapper (354), Swiss Target Prediction (100), STITCH (10), CTD (194), DGIdb (32), ECTM (72), and SymMap (64). We established a luteolin-related target set by syndicating a union of the predicted results and 638 targets related to luteolin were gathered after the removal of duplications and transferring gene symbols (**Figure 2A**).

**Figure 3:**
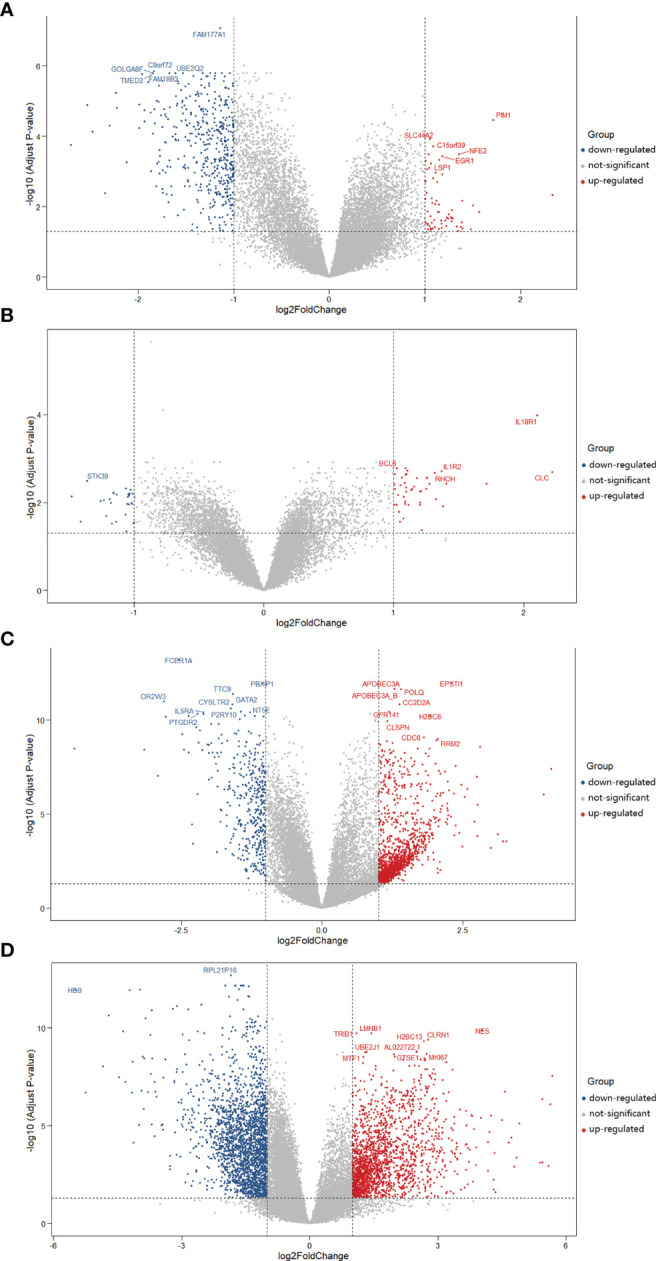
Volcano plots of differentially expressed genes (DEGs) for asthma and COVID-19 patients. The abscissa represented log2FC and the ordinate indicated the −log10 (adjusted *P*-value) of the genes. The red and blue points, respectively, represented the upregulated and downregulated genes with the criteria of adjusted *P*-value <0.05 and |log2FC| >1. **(A)** DEGs from GSE74986 were collected from 74 asthma patients and 12 healthy controls. **(B)** DEGs from GSE76262 were obtained from 118 asthma patients and 21 healthy donors. **(C)** DEGs from GSE152641 originated from 62 COVID-19 patients and 24 healthy controls. **(D)** DEGs from GSE171110 were obtained from 44 COVID-19 patients and 10 healthy donors.

Subsequently, we obtained the DEGs of asthma and COVID-19 from GEO *via* analyzing GSE74986 (522 targets), GSE76262 (71 targets), GSE152641 (1,896 targets), and GSE171110 (4,023 targets). GSE74986 recruited 74 asthma patients and 12 healthy controls to isolate from bronchial alveolar lavage cells, and the profile of GSE76262 collected from induced sputum cells comprised 118 participants with asthma and 21 healthy donors. Volcano plots of DEGs for asthma patients are shown in **Figures 3A, B**. GSE152641 information originated from the whole blood of 62 COVID-19 patients and 24 healthy controls, and GSE171110 contained the whole-blood gene expression profiles of 44 COVID-19 patients and 10 healthy donors. Volcano plots of DEGs for COVID-19-infected patients are shown in **Figures 3C, D**.

Then, we continued to collect target genes related to COVID-19 from seven open-source databases, namely, CTD (7,252), DisGeNET (1,843), DrugBank (344), GeneCards (1,566), PubChem (637), TTD (93), and NCBI Gene (214). After checking duplications, 12,642 target genes were obtained (**Figure 2B**). As for asthma-related genes, we obtained the targets from six open-source databases as follows: 1) CTD (36,036), 2) DisGeNET (2,096), 3) GeneCards (7,686), 4) OMIM (185), 5) TTD (149), and 6) NCBI Gene (1,000). A total of 38,710 target genes were achieved after the removal of duplications (**Figure 2C**).

Finally, we received 538 common target genes between COVID-19, asthma, and luteolin, which were analyzed by the Venn diagram tool and Microsoft Excel (**Figure 2D**). The 538 target genes were used to further screen the hub target genes to construct the PPI network for luteolin against COVID-19/asthma comorbidity.

### PPI Network Analysis

**Figure 4:**
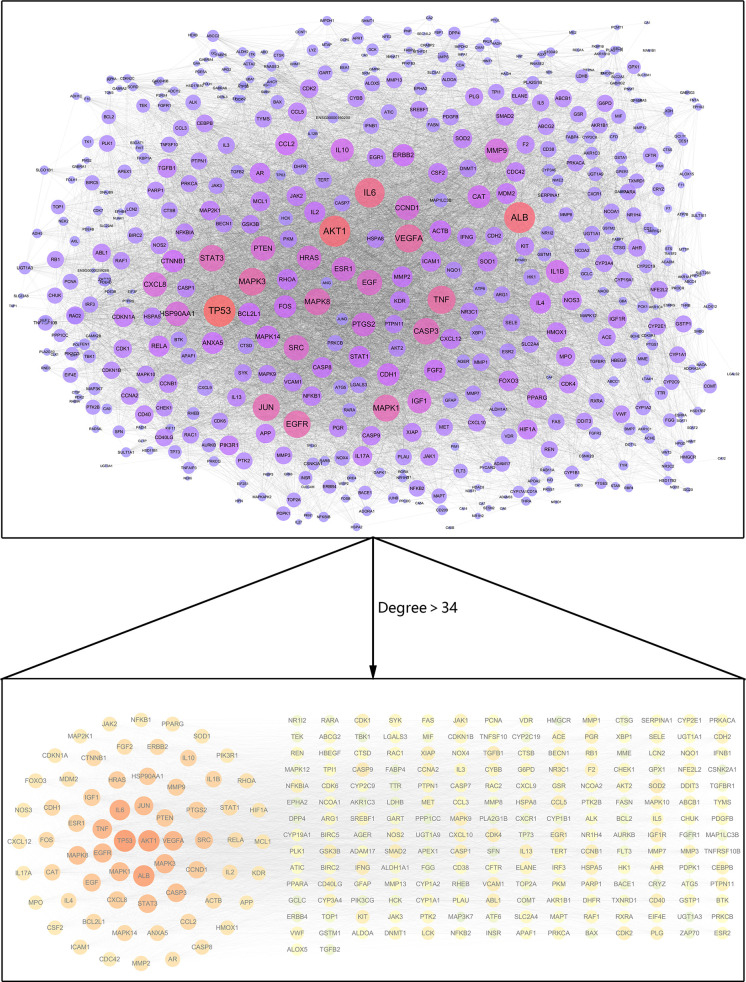
PPI network for hub targets of luteolin against COVID-19/asthma comorbidity. The nodes and edges, respectively, represented hub targets and interactions between targets. The sizes and color shades of nodes presented a positive correlation with degree values, and a node with a brighter color and larger shape played a more important role in fighting COVID-19/asthma comorbidity.

The interaction network between 538 common target genes was analyzed to screen the hub targets for luteolin against COVID-19/asthma comorbidity. The targets with degree values greater than the median were chosen as the hub targets, and the results showed that the median degree value was 34 and the targets with degree values greater than 34 were regarded as hub targets. Thus, a total of 264 hub targets were identified and the PPI network was constructed by the STRING 11.0b database and visualized by Cytoscape 3.7.2 software as shown in **Figure 4**. The nodes and edges, respectively, represented targets and interactions between targets, and there were 264 nodes and 8,967 edges in the PPI network of hub targets. In particular, the sizes and color shades of nodes presented positive correlation with degree values, indicating that a node with a darker color and larger shape plays a more important role in fighting COVID-19/asthma comorbidity. As seen in **Figure 4**, the top 6 targets with the highest degree values were TP53 (degree = 272), AKT1 (degree = 260), ALB (degree = 258), IL-6 (degree = 241), TNF (degree = 218), and VEGFA (degree = 218). Consequently, TP53, AKT1, ALB, IL-6, TNF, and VEGFA as the crucial players for luteolin to treat COVID-19/asthma comorbidity were further used to perform molecular docking with luteolin.

### GO Enrichment Analysis

**Figure 5:**
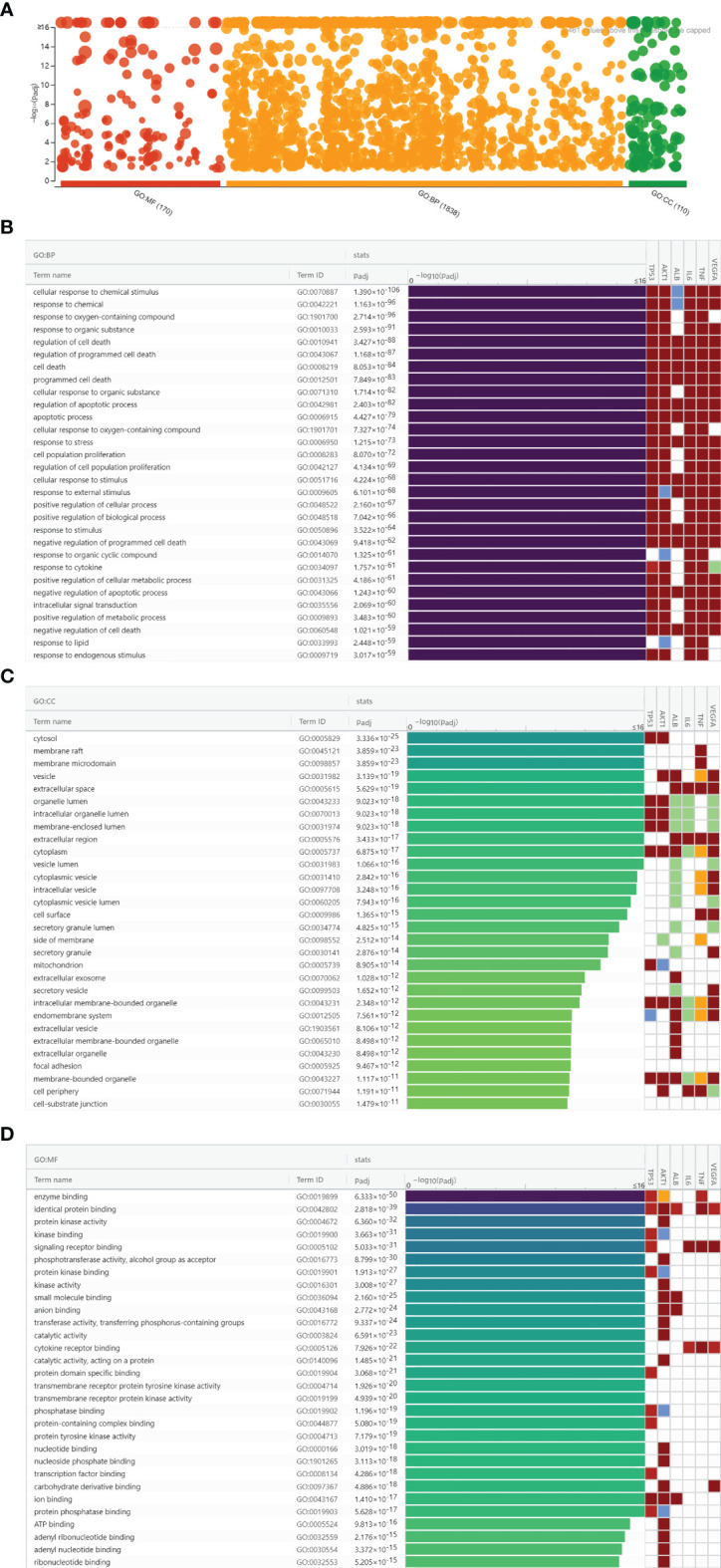
Gene Ontology enrichment analysis results of luteolin against COVID-19/asthma comorbidity. **(A)** The results of biological process (BP), cellular component (CC), and molecular function (MF) enrichment analyses. The abscissa indicated the kind of GO enrichment analyses, while the ordinate represented the −log10 (adjusted *P*-value) of the terms. The red, orange, and green points, respectively, represented the MF, BP, and CC enrichment analyses terms. **(B)** Identification result of BP terms according to adjusted *P*-value. **(C)** Identification result of CC terms according to adjusted *P*-value. **(D)** Identification result of MF terms according to adjusted *P*-value.

To further explore the biological functions of luteolin against COVID-19/asthma comorbidity, hub targets were submitted to g:Profiler for GO enrichment analysis. A total of 2,118 GO terms were obtained consisting of 1,838 BP terms, 110 CC terms, and 170 MF terms as shown in **Figure 5A**. The top 30 terms of BP, CC, and MF were ranked by adjusted *P*-value and the enrichment condition of TP53, AKT1, ALB, IL-6, TNF, and VEGFA are shown in **Figures 5B–D**. BP enrichment analysis mainly contained cellular response to chemical stimulus; response to chemical, immune process; response to oxygen-containing compound; and so on. CC enrichment analysis mainly contained cytosol, membrane raft, membrane microdomain, and so on. MF enrichment analysis mainly contained enzyme binding, identical protein binding, protein kinase activity, and so on. The results of GO terms suggested that luteolin may regulate the cell cycle process, immune response, oxidative stress, virus defense, and inflammatory response *via* the cytosol, membrane raft, and membrane microdomain to perform its therapeutic effects against COVID-19/asthma comorbidity.

### Pathway Enrichment Analysis

**Figure 6:**
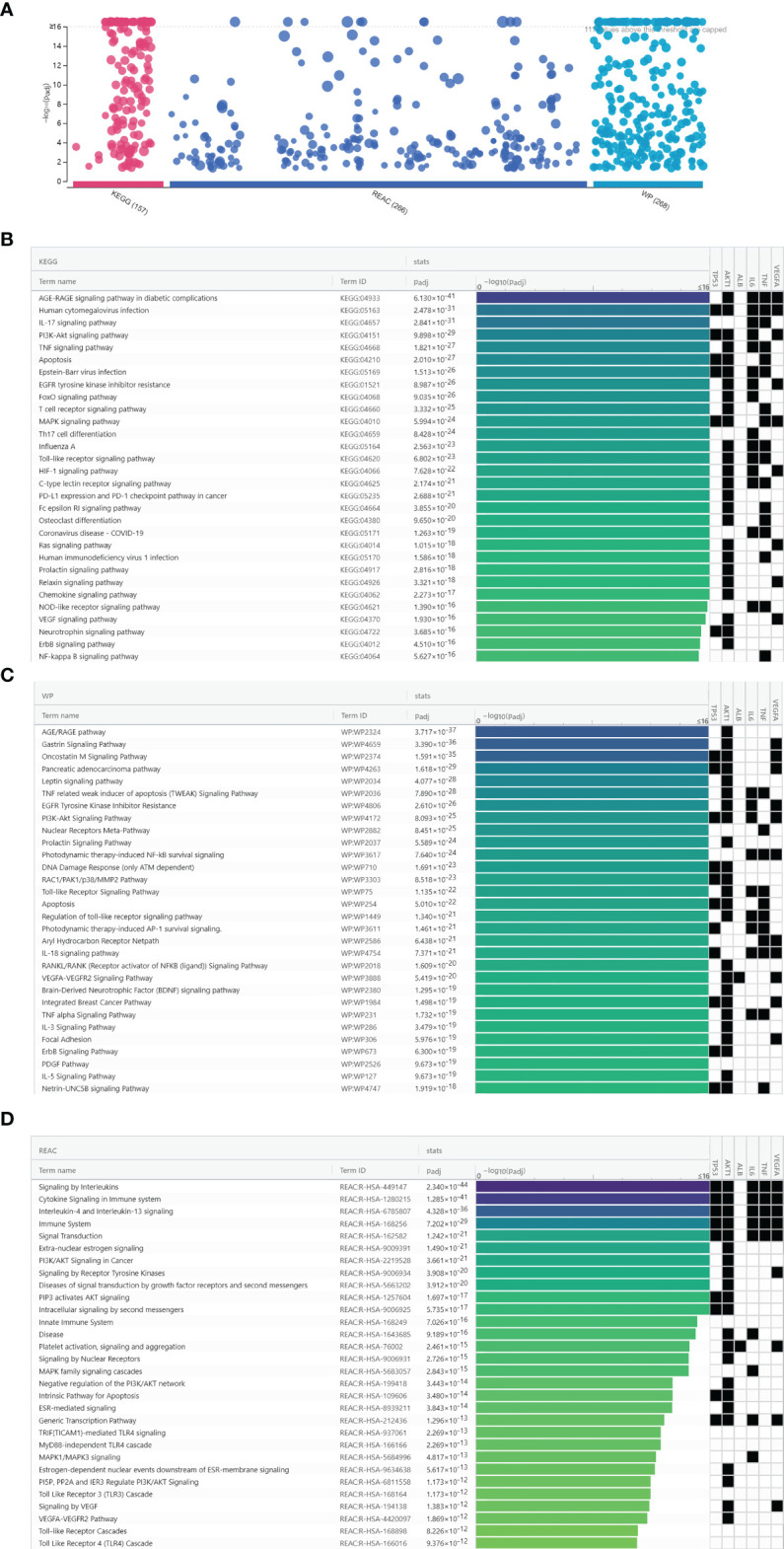
Biological pathway enrichment analysis results of luteolin against COVID-19/asthma comorbidity. **(A)** The results of KEGG, Wiki, and Reactome pathway enrichment analyses. The abscissa indicated the kind of biological pathway enrichment analysis, while the ordinate represented the −log10 (adjusted *P*-value) of the terms. The purple, dark blue, and light blue points, respectively, represented the KEGG, Reactome, and Wiki pathway enrichment analyses terms. **(B)** KEGG pathway enrichment analysis identification result according to adjusted *P-*value. **(C)** Wiki pathway enrichment analyses identification result according to adjusted *P*-value. **(D)** Reactome pathway enrichment analysis identification result according to adjusted *P*-value.

A total of 691 pathway terms were recognized consisting of 157 KEGG pathways, 266 Reactome pathways, and 268 Wiki pathways as shown in **Figure 6A**. The top 30 pathways were ranked by adjusted *P*-value and the enrichment condition of TP53, AKT1, ALB, IL-6, TNF, and VEGFA are presented in **Figures 6B–D**. KEGG pathways were mainly involved in the Toll-like receptor signaling pathway, AGE–RAGE signaling pathway in diabetic complications, human cytomegalovirus infection, IL-17 signaling pathway, and so on. Reactome pathways were significantly enriched in the AGE–RAGE signaling pathway, TNF signaling pathway, signaling by interleukins, cytokine signaling in immune system, IL-4 and IL-13 signaling, and so on. Wiki pathways predominantly included the AGE/RAGE pathway, MAPK signaling pathway, gastrin signaling pathway, oncostatin M signaling pathway, and so on. The enriched pathways of luteolin against COVID-19/asthma comorbidity were strongly associated with inflammation, immune response, regulation of vascular circulation, hypoxia, cell growth, and cell cycle process.

### Virus-Associated GO and Pathway Terms Enriched by the Hub Targets

Surprisingly, we found that three GO terms, namely, response to virus, cellular response to virus, and defense response to virus, were significantly enriched. Additionally, eight KEGG terms and four Wiki terms were significantly enriched including coronaviruses, Ebola virus pathway, human cytomegalovirus infection, herpes simplex virus, and so on. The above results suggest that luteolin may perform a broad antiviral effect through regulating these GO and pathways terms related to targets as exhibited in **Table 3**.

**Table 3:** Virus-related GO and pathway enrichment analyses terms.

Source	Term ID	Term name	Adjusted *P*-value		−Log10 (adjusted *P*-value)	Count
GO : BP	GO:0009615	Response to virus		6.27 × 10^−8^	7.20	26
GO : BP	GO:0098586	Cellular response to virus		3.63 × 10^−5^	4.44	11
GO : BP	GO:0051607	Defense response to virus		4.84 × 10^−3^	2.32	16
KEGG	KEGG:05167	Kaposi sarcoma-associated herpesvirus infection		6.55 × 10^−35^	34.18	54
KEGG	KEGG:05163	Human cytomegalovirus infection		2.48 × 10^−31^	30.61	54
KEGG	KEGG:05169	Epstein–Barr virus infection		1.51 × 10^−26^	25.82	47
KEGG	KEGG:05166	Human T-cell leukemia virus 1 infection		9.53 × 10^−24^	23.02	46
KEGG	KEGG:05171	Coronavirus disease—COVID-19		1.26 × 10^−19^	18.90	43
KEGG	KEGG:05170	Human immunodeficiency virus 1 infection		1.59 × 10^−18^	17.80	40
KEGG	KEGG:05165	Human papillomavirus infection		5.42 × 10^−15^	14.27	45
KEGG	KEGG:05168	Herpes simplex virus 1 infection		7.63 × 10^−4^	3.12	35
Wiki	WP : WP4864	Host–pathogen interaction of human coronaviruses—apoptosis		4.23 × 10^−12^	11.37	13
Wiki	WP : WP4877	Host–pathogen interaction of human coronaviruses—MAPK signaling		7.07 × 10^−10^	9.15	14
Wiki	WP : WP4880	Host–pathogen interaction of human coronaviruses—interferon induction		6.36 × 10^−8^	7.20	12
Wiki	WP : WP4217	Ebola virus pathway on host		2.33 × 10^−6^	5.63	20

### Binding Activities of Luteolin to COVID-19/Asthma-Related Targets

The results showed that luteolin had good binding activities (all binding energy < 0 and all RMSD < 2) with COVID-19 and the top 6 hub targets as shown in **Table 4**. Regarding COVID-19-associated target proteins, luteolin had the best binding activity with S-RBD and also showed promising affinities with Mpro, TMPRSS2, ACE2, CD147, and S-protein. Among the top 6 hub targets of luteolin against COVID-19/asthma comorbidity predicted by the PPI network, the results showed that binding activity of luteolin with TNF was the best, and AKT1, ALB, TP53, IL-6, and VEGFA performed compact binding patterns with luteolin, but secondary to TNF.

**Table 4:** Molecular docking results of luteolin with targets.

Number	Target protein	PDB ID	RMSD	Binding energy (kcal/mol)
A	S	6VSB	0.55	−5.10
B	RBD	6W41	0.43	−7.60
C	ACE2	1R42	0.34	−6.00
D	Mpro	6LU7	0.71	−7.30
E	TMPRSS2	7MEQ	0.50	−6.90
F	CD147	3B5H	0.00	−5.20
G	TP53	6GGA	0.00	−7.60
H	AKT1	3CQW	0.18	−8.30
I	ALB	2BX8	0.00	−8.30
J	IL-6	1ALU	0.85	−6.20
K	TNF	6OP0	0.04	−8.90
L	VEGFA	3QTK	1.39	−5.10

**Figure 7:**
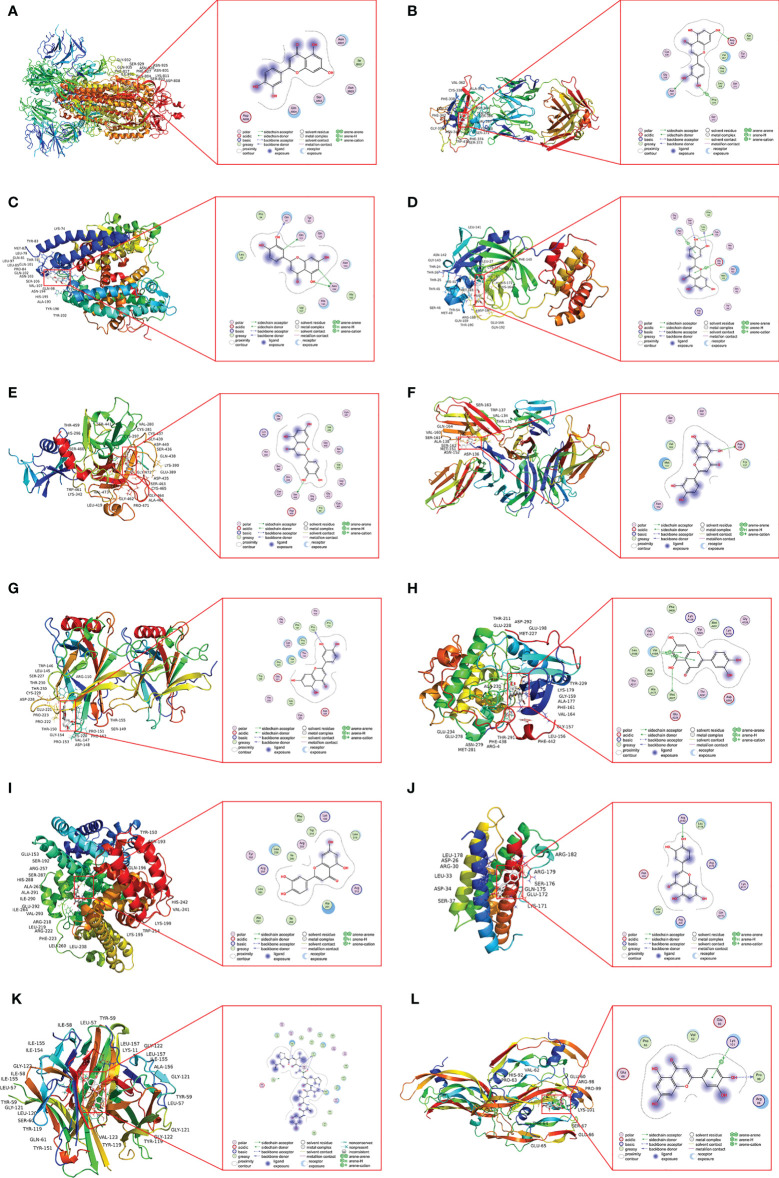
The docking model of luteolin with COVID-19 and the identified top 6 critical targets. **(A)** Docking result of luteolin and S-protein. **(B)** Docking result of luteolin and RBD protein. **(C)** Docking result of luteolin and ACE2 protein. **(D)** Docking result of luteolin and Mpro protein. **(E)** Docking result of luteolin and TMPRSS2 protein. **(F)** Docking result of luteolin and CD147 protein. **(G)** Docking result of luteolin and TP53 protein. **(H)** Docking result of luteolin and AKT1 protein. **(I)** Docking result of luteolin and ALB protein. **(J)** Docking result of luteolin and IL-6 protein. **(K)** Docking result of luteolin and TNF protein. **(L)** Docking result of luteolin and VEGFA protein.

Luteolin and ACE2 protein formed hydrogen bonds at the amino acid residues Gln 81, Gln 101, and Asn 194. It was shown that luteolin was linked to Ser 436 of the TMPRSS2 protein by hydrogen bonding. Luteolin was also attached to Asn 343 and Asp 364 of RBD protein by hydrogen bonds and Phe 374 of RBD protein *via* π–H bonds. Similarly, luteolin was connected to Ser 144, Leu 141, and Cys 145 of Mpro protein through hydrogen bonds and Asn 142, Glu 166, of Mpro protein by π–H bonds. Luteolin formed a hydrophobic interaction with Gln A804 and Asn A801 of S-protein. The Pro 152 of TP53 protein, the Arg B182 of IL-6 protein, and Ala C156, Ile C154, and Phe C152 of TNF protein formed hydrogen bonds with luteolin. The AKT1 protein not only formed hydrogen bonds with luteolin at Met A227 but also formed π–H bonds with it at Val A164. Luteolin was connected to Pro 99 of VEGFA protein by hydrogen bonds and connected to Lys 101 through π-cation bonds. Luteolin and ALB protein constituted hydrophobic interaction at Ala 291 and Leu 238 (**Figures 7A–L**).

## Discussion

We acquired 264 hub targets of luteolin against COVID-19/asthma comorbidity. GO analysis predicted that luteolin has the potential to regulate the cell cycle process, immune response, oxidative stress, inflammatory response, and virus defense to perform its therapeutic effects. Pathway analysis suggested that luteolin may regulate the Toll-like receptor signaling pathway, MAPK signaling pathway, TNF signaling pathway, and so on to combat COVID-19/asthma comorbidity. Moreover, molecular docking verified that luteolin and the top 6 genes and proteins related to COVID-19/asthma comorbidity showed good binding activities. Therefore, the findings illustrate that luteolin with promising drug likeness and biological activities has great potential to become a beneficial therapeutic approach for COVID-19/asthma comorbidity.

### The Ideal Physicochemical Properties and Bioactivity of Luteolin

It is well accepted that a compound that complies with the Lipinski’s rule of five tends to have favorable pharmacokinetic properties and further improves the possibility of evolving into a drug candidate (65, 66). TPSA comprises intestinal absorption, bioavailability, permeabilization of Caco-2, and blood–brain barrier penetration, and TPSA value of oral drugs should not exceed 140 Å (37, 62–64). It is considered that a compound with physicochemical properties of TPSA value of ≤140 Å and *n*-rotb ≤10 has a bigger potential to show good oral bioavailability (37). In addition, %ABS value in light of the formula %ABS = 109 − [0.345 × TPSA] is considered to penetrate the cell membranes well when the %ABS value ranges from 57.95% to 78.98% (38, 67). To sum up, *in-silico* drug likeness and ADME (including absorption, distribution, metabolism, and excretion) are evaluated according to the following aspects: Lipinski’s rule of five, TPSA, and %ABS. The results showed that luteolin fitted with the criteria of milogP = 1.97 < 5, MW = 286.24 < 500 Da, *n*-ON = 6 < 10, and *n*-OHNH = 4 < 5 without violation of the simplified Lipinski’s rule of five. Furthermore, the value of TPSA at 111.12 Å < 140 Å and %ABS at 70.66 that ranged from 57.95% to 78.98% were at the range of ideal oral bioavailability. These results reveal that luteolin theoretically would not have caveats about drug likeness and ADME.

Studies reveal that luteolin can be absorbed with plasma concentrations reaching the highest peak level at 1.1 h and the oral bioavailability reaches 26% ± 6% (68, 69). Luteolin and glycosylated forms may be converted into sulfated, methylated, and/or glucuronidated metabolites through phase II metabolism and then enter into the systemic circulation or return to the small intestine by enterohepatic cycling (70). The solubility of the luteolin–phospholipid complex in water is about 2.5 times higher and has bioavailability improvement compared with luteolin, indicating that transformation of luteolin delivery can increase the absorption (71).

A molecular compound with a bioactivity value of >0.00 has a great possibility to exert great biological activity, while a bioactivity score between −0.50 and 0.00 is considered to moderately perform biological activity and bioactivity value of ≤0.50 is perceived as poor biological activity (37, 39, 63, 64). The results of bioactivity analysis showed that the pharmacological effects of luteolin theoretically involve several mechanisms, including excellent interactions with kinase inhibitor, nuclear receptor ligand, and enzyme inhibitor and moderate interactions with GPCR ligands, ion channel modulator, and protease inhibitor. The above results illustrate that luteolin is expected to perform pharmacological effects *via* the five mechanisms listed above with relatively good biological activity scores.

### Luteolin Might Trigger Hub Targets to Fight Against COVID-19/Asthma Comorbidity

We first obtained 538 common targets of the luteolin and the COVID-19/asthma, and 264 targets with degree values greater than the median at 34 in the PPI network were selected as the hub targets. Moreover, we chose the top 6 targets in the PPI network and critical proteins related to COVID-19 to perform molecular docking with luteolin. The top 6 target genes included TP53 > AKT1 > ALB > IL-6 > TNF > VEGFA, and the other relatively hub targets comprised CASP3, IL-4, MAPK3, EGFR, MAPK1, MAPK8, STAT3, IL-10, CXCL8, IL-1β, IL-17A, ACE, and so on.

SARS-CoV-2 infection may induce lymphocyte apoptosis by promoting the activation of TP53 to regulate immune inflammatory response (72, 73). ACE2 can affect TP53 expression in lung endothelial cells and TP53 binding site deletions lead to the increase in promoter activity of ACE2 (74, 75). A study collects blood samples and finds that the expression of TP53 increases in COVID-19 patients compared with healthy controls, although it is not statistically significant (73). Alternatively, TP53 methylation is strongly related to the severity of asthma and the genetic polymorphism of TP53 contributes to asthma susceptibility (76, 77). Overexpressed AKT1 facilitates viral protein synthesis and AKT1 silencing contributes to viral RNA expression reduction, inhibition of viral capsid protein synthesis, and virus release (78, 79). Consistent with our prediction, a study confirms that inhibition of AKT1 reduces viral yields in Huh7 cells infected by SARS-CoV-2 (80). AKT1 promotes the airway myocyte hypertrophy and is observed to be activated in asthmatic subjects, which might lead to airway smooth muscle hyperplasia involved in asthma exacerbations (81, 82). Additionally, AKT1 is involved in regulating the immune function and activated phenotype of macrophages by regulating innate immunity, and AKT1 activation intensifies inflammation and metabolism-related responses (83, 84). ALB is an important protein for maintaining nutrition of the body and a normal nutritional status is an essential element for the immune system to fight against infection and inflammation (85). A low level of ALB is closely related to poor survival of COVID-19, and asthmatic children show decreased ALB compared with healthy control involved in increased FeNO (86, 87). The levels of VEGFA involved in coagulopathy and thrombosis are significantly elevated, indicating that the condition of hypoxemia and inflammation exist in COVID-19 patients (88, 89). It is well known that inhibition of VEGF helps to improve abnormal angiogenesis and vascular leakage to reduce airway vascular remodeling and airway mucus density in asthma (90). Apoptosis has been considered as an important defense against inflammation involved in antiviral and anti-asthma effects, and the level of CASP3 represents the degree of caspase-dependent apoptosis (91, 92).

The activation of MAPK family members and MAPK–STAT3 axis leads to overexpression of inflammatory factors including IL-1β, TNF-α, and IL-6 (93). TNF is an important proinflammatory cytokine involved in the immune process, and highly expressed IL-6 leads to an increase in neutrophils and a decrease in lymphocytes, which makes the inflammatory response more severe and thus takes an important position in inducing the cytokine storm (94). SARS-CoV-2 is proven to promote IL-6, TNF, and IL-10 expression by stimulation of macrophages or spleen or lymph nodes *via* binding to ACE2, resulting in a decrease in lymphocytopenia involved in the immune imbalance and cytokine storm (95, 96). Transcriptional analysis confirms that CXCL8 levels significantly show an upward trend in COVID-19 patients, which triggers the recruitment of neutrophils to aggravate the inflammatory injury (73). Researches show that the levels of IL-17, IL-1β, TNF‐α, IL-6, and IL-4 significantly increase in COVID-19 patients compared with non-COVID-19 patients or healthy people, and IL-17, IL-1, and TNF have great dependence on Th17 adaptive immune response triggering the inflammation cascades in COVID-19 (26, 94, 97, 98). Asthma is characterized by chronic inflammatory infiltration caused by inflammatory cells and immune cells, and inhibition of TNF-α, IL-1β, IL-17, IL-4, and IL-6 contributes to improving asthma (99). Furthermore, biomarkers of MAPK family members and JAK/STAT signaling pathways show a significant upward trend in asthma patients (100).

Surprisingly, a study proves that luteolin significantly inhibits the expression of TP53 (101). Another study confirm that luteolin can block AKT signaling to balance immune response and reduce inflammatory injury to improve prognosis during COVID-19/asthma comorbidity (102). Research reveal that luteolin interacts with kinds of amino acid residues of subdomain IIA making the ALB structure more stable (103). Moreover, luteolin has been shown to perform promising anti-inflammatory and immune regulatory effects through decreasing the expression of IL-1β, IL-6, MAPK family, TNF-α, STAT3, and IL-17 (104–106). Furthermore, molecular docking confirmed that luteolin showed ideal binding activities with hub targets including TP53, AKT1, ALB, IL-6, TNF, and VEGFA. These binding results reveal that luteolin may likely bring TP53, AKT1, ALB, IL-6, TNF, and VEGFA to target COVID-19/asthma comorbidity. ACE2 and CD147 are the dominant receptors for viral entrance into host cells and TMPRSS2 is the main non-endosomal pathway for viruses to enter the cells (107). We found that luteolin can efficiently bind to ACE2, Mpro, S, S-RBD, TMPRSS2, and CD147, suggesting that luteolin may directly target the novel coronavirus to perform antiviral function. To summarize, we believe that luteolin has the great potential to help increase the treatment effect of present clinical antiviral approaches and immunotherapy to treat the lethal COVID-19 or COVID-19/asthma comorbidity.

### The Critical Mechanisms for Luteolin to Combat COVID-19/Asthma Comorbidity

The GO results showed that BP was enriched in virus defense, defense and regulation of inflammation, immune responses, oxidative stress, cell growth, and cell replication. On the one hand, the MF results revealed that luteolin may perform pharmacological treatment for COVID-19/asthma comorbidity through interaction with multiple proteases, signaling receptor, nuclear receptor, small molecule, and ribonucleotide. The results of MF were consistent with the biological activity scores prediction, further indicating that luteolin may hold great potential to exert a therapeutic function for COVID-19 and the duration of asthma *via* a diversity of mechanisms. We further confirmed that anti-COVID-19 and anti-asthma effects performed by luteolin were mainly directed through immunomodulatory, antioxidant, antiviral, and anti-inflammatory signaling pathways, including Toll-like receptor signaling pathway, MAPK signaling pathway, PD-L1 expression and PD-1 checkpoint pathway in cancer, TNF signaling pathway, apoptosis, PI3K–AKT signaling pathway, EGFR tyrosine kinase inhibitor resistance, ErbB signaling pathway, HIF-1 signaling pathway, AGE/RAGE signaling pathway, and so on.

Toll-like receptor signaling pathways are identified as key factors that are responsible for regulating immune defense mechanisms against pathogenic microorganisms. It is well recognized that most Toll-like receptor signaling pathways share similar signal transduction pathways through MyD88-dependent pathways, which involve NF-κB and JAK/STAT signaling pathways to activate the production of various cytokines (IL-6, IL-1β, TNF-α, and so on), which is strongly associated with the progress of asthma and COVID-19 (108–111). Activation of the PI3K–AKT signaling pathway is associated with various biological processes, including cell cycle, apoptosis, metabolism, and angiogenesis (112). It has been revealed that CD147 activation leads to overexpression of the PI3K/AKT signaling pathway involved in SARS-CoV-2 endocytosis (107, 113). Suppression of MAPK and PI3K/AKT signaling pathways helps to regulate the differentiation of T cells to improve inflammatory response in asthma (114). EGFR, a tyrosine kinase receptor that is required for the activation and proliferation of inflammatory cells, is critical for dictating clinical manifestations in asthma and COVID-19 (115, 116). *In-vitro* experiments suggest that the expression of key regulators in ErbB, MAPK, AKT/mTOR, and TNF signaling pathways related to cell proliferation, inflammatory response, immune response, oxidative stress, and apoptosis significantly increases in Huh7 cells infected by SARS-CoV-2 (80). However, HIF-1α expression significantly decreases during SARS-CoV-2 infection, and the absence of HIF-1α results in increased viral replication and severe inflammation (117). It is worth noting that the TNF signaling pathway has a strong association with the HIF-1 signaling pathway, manifested in activating AKT and MAPK signaling pathways to induce HIF-1α expression (118). AGEs can elevate the degree of oxidative stress by combining with RAGE, and RAGE is only highly expressed in the lungs, leading to the overexpression of proinflammatory mediators and excess inflammatory responses (119, 120). PD-1, a member of the B7/CD28 family, triggers a series of intracellular signal transduction leading to T-cell suppression and exhaustion when binding to PD-L1 and PD-L2 (121, 122). Specifically, it is confirmed that the levels of PD-1 significantly increase in COVID-19 patients and present a positive correlation with COVID-19 severity, and the study reveals that targeting PD-1 has therapeutic potential in treating COVID-19 and asthma (121, 122).

Luteolin can exert a broader range of anti-inflammatory and antioxidant effects and also play a critical role in regulating immune function and vascular circulation (106, 123). According to previous findings and the predicted results in our study, the key molecular mechanisms of luteolin against COVID-19/asthma comorbidity are summarized as follows. First, luteolin has broad antiviral activities and can specifically target the proteins required for COVID-19 infection and may exert an antiviral effect through multiple mechanisms as the targets predicted in our study (25, 26). Second, luteolin with potent anti-inflammatory, immunoregulatory, and antioxidant effects can inhibit inflammatory cascade to control the “cytokine storm” through decreasing the expression of various inflammatory mediators, including IL-1β, IL-6, MAPK family, TNF-α, STAT3, IL-17, and so on (104–106). Third, luteolin can improve vascular circulation *via* decreasing the vascular inflammation caused by activation of NF-κB and TNF-α and has the great potential to improve abnormal angiogenesis and vascular leakage through targeting VEGFA and other relevant mechanisms (88–90, 123).

## Conclusions

In conclusion, the findings from system pharmacology and bioinformatics analysis emphasized that antiviral, anti-inflammatory, antioxidant, and immunomodulatory effects and regulation of blood circulation were identified as crucial targets/pathways of luteolin against COVID-19/asthma comorbidity. Furthermore, luteolin with promising physicochemical properties and bioactivity may be clinically used to treat COVID-19 or COVID-19/asthma comorbidity based on the predicted biological processes and pharmacological mechanisms. Moreover, the potential and critical pharmacological targets of luteolin against COVID-19/asthma comorbidity provide the direction for further study, but the predicted results still need to be rigorously verified. In the next study, we intend to use the new coronavirus to infect macrophage cell lines and airway epithelial and alveolar epithelial cells to mimic the state of COVID-19/asthma comorbidity. Moreover, we will further analyze the mechanism of luteolin against COVID-19/asthma comorbidities through proteomics, genomics, metabolomics, and proteomics.

## Data Availability Statement

The datasets presented in this study can be found in online repositories. The names of the repository/repositories and accession number(s) can be found in the article/supplementary material.

## Author Contributions

X-FH, S-FZ, and X-HL conceived and designed this research. Y-ZX wrote the manuscript and participated in the design of the study. Y-ZX and Z-QS were responsible for the bioinformatics analysis and network construction. C-WP and H-TH carried out the data analysis and data interpretation. Z-QS is responsible for part of the network analysis of the article. All authors contributed to the article and approved the submitted version.

## Funding

This work was supported by the National Natural Science Foundation of Guangdong, China (Grant no. 2020A1515010589 and Grant no. 2021A1515010146), and the Science and Technology Program of Guangzhou, China (Grant no. 201904010235). This work was also supported by the “Double First-Class” and High-Level University Discipline Collaborative Innovation Team Project of Guangzhou University of Chinese Medicine (Grant no. 2021XK16), Guangdong Provincial Department of Education Innovation Team Project (Grant No. 2018KCXTD007), Key-Area Research and Development Program of Guangdong Province (Grant no. 2020B1111100002), National Natural Science Foundation of China (Grant no. 81973814 and no. 81904132), and the Technology Research of COVID-19 Treatment and Prevention and Special Project of Traditional Chinese Medicine Application-Research on the platform construction for the prevention and treatment of viral infectious diseases with traditional Chinese medicine (Grant no. 2020KJCX-KTYJ-130).

## Conflict of Interest

The authors declare that the research was conducted in the absence of any commercial or financial relationships that could be construed as a potential conflict of interest.

## Publisher’s Note

All claims expressed in this article are solely those of the authors and do not necessarily represent those of their affiliated organizations, or those of the publisher, the editors and the reviewers. Any product that may be evaluated in this article, or claim that may be made by its manufacturer, is not guaranteed or endorsed by the publisher.
